# Determination of Zinc-Based Additives in Lubricating Oils by Flow-Injection Analysis with Flame-AAS Detection Exploiting Injection with a Computer-Controlled Syringe

**DOI:** 10.1155/JAMMC.2005.1

**Published:** 2005

**Authors:** Gustavo Pignalosa, Moisés Knochen, Noel Cabrera

**Affiliations:** 1 Departamento “Estrella Campos,” Facultad de Química Universidad de la República Av. Gral. Flores 2124,Casilla 1157 Montevideo 11800 Uruguay

## Abstract

A flow-injection system is proposed for the determination of metal-based additives in lubricating oils. The system, operating under computer control uses a motorised syringe for measuring and injecting the oil sample (200 μL) in a kerosene stream, where it is dispersed by means of a packed mixing reactor and carried to an atomic absorption spectrometer which is used as detector. Zinc was used as model analyte. Two different systems were evaluated, one for low concentrations (range 0–10 ppm) and the second  capable of providing higher dilution rates for high concentrations (range 0.02%–0.2% w/w). The sampling frequency was about 30 samples/h. Calibration curves fitted a second-degree regression model (*r* ^2^ = 0.996). Commercial samples with high and low zinc levels were analysed by the proposed method and the results were compared with those obtained with the standard ASTM method. The *t* test for mean values showed no significant differences at the 95% confidence level. Precision (RSD%) was better than 5% (2% typical) for the high concentrations system. The carryover between successive injections was found to be negligible.


Journal of Automated Methods & Management in Chemistry, **2005** (2005), no. 1, 1–7
© 2005 Hindawi Publishing Corporation

### Determination of Zinc-Based Additives in Lubricating Oils by Flow-Injection Analysis with Flame-AAS Detection Exploiting Injection with a Computer-Controlled Syringe

Gustavo Pignalosa, Moisés Knochen, and Noel Cabrera

*Departamento "Estrella Campos," Facultad de Química, Universidad de la República, Av. Gral. Flores 2124, Casilla 1157, 11800 Montevideo, Uruguay*

*Received 10 August 2004; Accepted 24 August 2004*

A flow-injection system is proposed for the determination of metal-based additives in lubricating oils. The system, operating under computer control uses a motorised syringe for measuring and injecting the oil sample (200 $\mu$L) in a kerosene stream, where it is dispersed by means of a packed mixing reactor and carried to an atomic absorption spectrometer which is used as detector. Zinc was used as model analyte. Two different systems were evaluated, one for low concentrations (range 0–10 ppm) and the second capable of providing higher dilution rates for high concentrations (range 0.02%–0.2% w/w). The sampling frequency was about 30 samples/h. Calibration curves fitted a second-degree regression model ($r^2 = 0.996$). Commercial samples with high and low zinc levels were analysed by the proposed method and the results were compared with those obtained with the standard ASTM method. The $t$ test for mean values showed no significant differences at the 95% confidence level. Precision (RSD%) was better than 5% (2% typical) for the high concentrations system. The carryover between successive injections was found to be negligible.

### 1. INTRODUCTION

Lubricating oils consist of a base of mineral or synthetic oil and several substances added in order to enhance different properties of the product [1]. Some of these additives are salts of organic acids and metals such as calcium, barium, magnesium, and zinc. Depending on the additive and the characteristics of the oil, metal concentrations range typically from 0.2 to 2 g/L. The concentration of the additives should be determined, either as a part of the quality control of the final products, or to provide information about the oil during its life cycle. This determination is carried out, according to standard methods from the American Society for Testing and Materials (ASTM), by means of either inductively coupled plasma optical-emission spectrometry (ICP-OES) [2, 3] or flame atomic absorption spectrometry (FAAS) [4, 5]. These techniques are also used for the determination of wear metals in used lubricating oils, which is an activity carried out within predictive/proactive maintenance schemes of large engines and lubricated machinery.

From the analytical point of view, lubricating oils are a difficult matrix due to their high viscosity and hydrophobicity, which precludes direct introduction to standard nebulisers employed in ICP-OES and FAAS, as well as dilution with aqueous solvents. Thus, instrumental determinations usually require a dilution with organic solvent, for instance xylene, methyl-isobuthyl ketone, or kerosene. The samples are prepared by weight to avoid undesired volume uncertainties due to the viscosity of the oil. Organometallic metal standards are dissolved and diluted as necessary in the same solvent.

In order to avoid the need of organometallic standards, Wittmann [6] and Hon et al. [7] proposed the use of aqueous metal standards and an appropriate solvent chosen to allow the dilution of these standards.

These procedures, as well as the standard methods mentioned before, share some drawbacks. All of them require intensive hand labour and large amounts of glassware are needed for performing the dilutions. This glassware should then be cleaned and prepared for future use.

The introduction of automation in the analytical laboratory [8, 9] permits several improvements, such as a reduction in the handling of samples and glassware, less solvent consumption, and reduced chemical wastes. However, the literature is rather scarce in automated methods for the analysis of oils. Flow-injection analysis (FIA) [10] has been the technique selected by several authors for this purpose, for the determination of the contents of either metal-based additives or wear metals. For instance, Granchi et al. [11] proposed the use of an FIA-based system for the determination of metals in lubricating oils by ICP-OES, however the heavy task of sample preparation was carried out by a laboratory

Correspondence and reprint requests to Moisés Knochen, Departamento "Estrella Campos," Facultad de Química, Universidad de la República, Av. Gral. Flores 2124, Casilla 1157, 11800 Montevideo, Uruguay; E-mail: mknochen@fq.edu.uy.

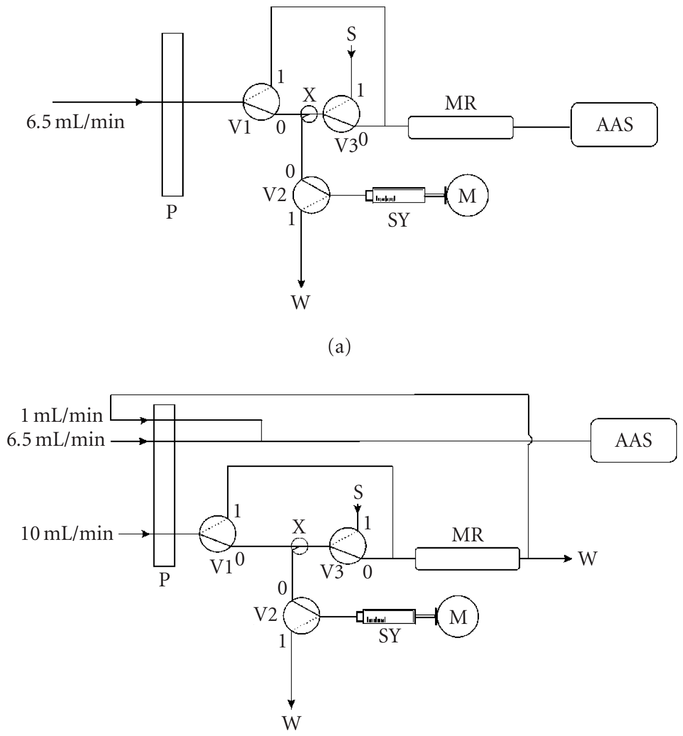

(a)

| Valves | Purge | Load | Inject |
|---|---|---|---|
| V1 | 1 | 1 | 0 |
| V2 | 1 | 0 | 0 |
| V3 | 1 | 1 | 0 |

(b)

Figure 1: Schematic diagram of (a) the low-concentration system (LCS) and (b) the high-concentration system (HCS). P denotes peristaltic pump, V1, V2, V3 three-way solenoid valves (“0”: default position; “1”: energised position), X mixing device, S sample, SY syringe, M syringe motor, W waste, MR mixing reactor, and AAS atomic absorption spectrometer.

robot, reducing the flow-injection system to the secondary task of sample introduction to the ICP spectrometer. A work by Borja et al. [12] suggested that FIA could be used for the determination of calcium-based additives in lubricating oils by means of emulsions and flame AAS detection. Online emulsification in an FIA system has also been proposed by Burguera et al. [13] for the automated determination of chromium in lubricating oils with electrothermal AAS detection.

Pignalosa and Knochen [14] reported the use of a flow-injection system for the determination of wear metals in lubricating oils, where injection of the sample was performed by a motorised syringe under computer control. This work demonstrated that syringe injection provides a robust means for accurately handling highly viscous oil samples.

On the other hand, metal concentration levels due to additives in the oil are usually much higher than those due to wear of the engine, thus the system must be capable of performing the necessary dilutions so that the final metal concentration in the sample bolus introduced to the nebuliser of the spectrometer is within a suitable range from the spectrometric point of view.

The objective of the present work is to explore the use of flow-injection analysis with syringe injection coupled to flame AAS as a way for automation of the determination of additives in lubricating oils in the routine quality control laboratory.

Zinc was chosen as a model analyte, considering that many lubricating oils contain high concentration of zinc-based additives. On the other hand, there is a group of lubricating oils intended for use in special engines containing parts with silver alloys. These oils should not contain zinc as the presence of this element may damage the engine. For both reasons, the development of an automated method for the determination of zinc is necessary in quality control laboratories, and the same approach can be used for the determination of additives containing other metals.

### 2. EXPERIMENTAL

### 2.1. *Apparatus*

The flow-injection system (Figure 1) was developed around a lab-made motorised syringe, built from a Hewlett-Packard

(Palo Alto, USA) 1 mL gas-tight syringe, driven by a stepping motor and mechanism taken from a dismantled large diskette drive. The stepping motor employed had a resolution of 1.8°/step, attaining a theoretical volumetric resolution of 2 $\mu$L with a maximum dispensing volume of about 450 $\mu$L. On start-up, the "zero" position of the syringe was initialised with the help of an optocoupler used as optic position sensor, and the number of steps was counted from this original position.

Three 3-way 12-volt solenoid valves (model HP225T031, NResearch, West Caldwell, USA) provided the necessary fluid switching.

The carrier fluid was driven by a Dynamax RP-1 peristaltic pump (Rainin Instrument Co., Woburn, USA) fitted with Viton tubing.

A lab-built mixing device (labelled "X" in Figure 1) and a packed mixing-reactor (labelled "MR" in the same figure), both already described elsewhere [14], were used to mix the streams of carrier and oil sample. The mixing device was machined in acrylic material and fitted with PTFE connectors (Omnifit, Cambridge, UK). The sample conduit was bored at an angle of 30°, respect to the carrier conduit, as previous experiments suggested that use of this angle produced less memory effects. The packed mixing reactor was constructed from a 6.0 cm length of 2.48 mm ID PTFE tubing and packed with small pieces of PTFE cut with a sharp blade from the same stock tubing. These pieces were basically 1 mm thick tubing slices in turn cut into smaller pieces. This reactor was fitted with PTFE connectors (Omnifit).

Detection was performed by a Perkin-Elmer (Norwalk, USA) model 380 FAAS spectrometer with 10 cm burner and air-acetylene flame, fitted with a Photron (Narre Warren, Australia) zinc hollow cathode lamp and operated at the wavelength of 213.9 nm. In the system for high concentrations (see below), the burner was rotated as necessary to decrease the absorbance values to appropriate levels.

The operation of the system, data acquisition, and control were carried out by means of an 80 MHz IBM-compatible 80486-based personal computer fitted with a multipurpose data acquisition and control board (CIO-DAS-08AOH, ComputerBoards, Middleboro, USA) installed on the ISA bus. The 12 bit analogue-to-digital converter (ADC) in the card was used to capture analogue data from the spectrometer's recorder output, while several of the digital input and output (I/O) ports were used for logic control of the stepping motor and solenoid valves. On-board counters were used to provide appropriate timing when needed.

A special lab-built control system supplied the necessary power to the electronic devices, motor, and solenoid valves, and processed the signals to and from the multipurpose ADC board. The overall connection and signal flow schematics is depicted in Figure 2.

Software compiled in QuickBASIC 4.0 (Microsoft) was developed for the operation of the system and run under the MS-DOS 6.0 operating system. The program controlled the operation of the stepping motor and solenoid valves, triggered data acquisition via the ADC, and scaled the raw data transferred from the ADC to obtain absorbance values which were plotted on-screen as a function of time and stored on hard disk for further processing.

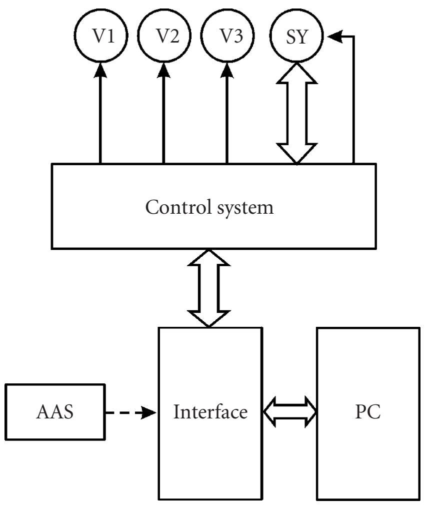

Figure 2: Schematics of connections and signals flow. V1, V2, V3 denote solenoid valves, SY motorised syringe, AAS atomic absorption spectrometer, PC personal computer, — power line, and - - - analog signal.

Data was saved to disk as ASCII file and processed post run by means of a chromatography program (Peak Simple II, version 3.3, SRI Inc., Torrance, USA).

A model 80550-20 strip-chart recorder (Cole-Parmer, Vernon Hills, USA) and a CR-6 recorder/integrator (Shimadzu, Kyoto, Japan) were also used for recording absorbance signals as a function of time.

### 2.2. *Flow systems*

In order to handle samples containing both low and high concentrations of zinc, two different systems were developed. The first system (Figure 1a) was used in the determination of traces of zinc in oils supposed to be zinc-free. This system was used with Zn concentrations below 10 ppm and will be called "low-concentration system" (LCS). A second system (Figure 1b) was designed for the determination of high concentrations of zinc in those oils containing zinc-based additives ("high-concentration system" or HCS). In these oils, zinc concentrations as high as 0.15%( w/w) can be found, making it necessary to implement an additional on-line dilution scheme. This system was used with Zn concentrations in the range 0.02%–0.2% (w/w).

The operation of the two systems was similar, the main difference being the split-flow and confluence scheme used in the second system (Figure 1b) in order to produce further dilution with carrier of the sample bolus. This scheme corresponds to the classical cascade dilution system proposed by Whitman and Christian [15].

The motorised syringe (SY) could be connected either to waste or to the flow system via a three-way solenoid valve (V2). When sample had to be loaded, a special purge cycle was performed. For this purpose valves V1 and V3 were energised and carrier flowed to the AAS spectrometer by an alternate pathway. The syringe was driven backwards and sample was loaded into the syringe passing through V3 and the mixing device X, flushing the pathway with fresh sample and eliminating the rest of carrier or previous samples. When the appropriate volume of sample (300 $\mu$L) was reached, the syringe stopped, valve V3 was energised and the contents of the syringe expelled to waste. This purge cycle was carried out two times in order to minimise carryover effects. Afterwards, the syringe was loaded with 200 $\mu$L of sample, the three valves were turned off, and the syringe was driven forward, thus injecting the sample at a rate of 2.4 mL/min into the flow system via the mixing device X, the solenoid valve V3, and the packed mixing reactor MR. From the output of this reactor, the dispersed sample bolus was either carried directly to the spectrometer's nebuliser (low-concentration system, Figure 1a) or submitted to further dilution with carrier via the splitting and confluence before sending it to the spectrometer (high-concentration system, Figure 1b).

### 2.3. Reagents, standards, and samples

The carrier was deodorised kerosene (ANCAP, Montevideo, Uruguay), which was used also as solvent in the reference (batch) method. This solvent was tested to be free of zinc.

Conostan 5000 ppm zinc in oil standard (Conoco Specialty Products Inc., Ponca City, USA), diluted as necessary with BO-75 base oil (Conoco), was used for calibrations.

Two groups of commercial lubricating oil samples, respectively with and without zinc-based additive, were used. Samples with additive were Rimula D 30, Helix Super 15W40, Tellus 46 (all from Shell), Superdiesel 40 (ANCAP), and GTS-99L1 and GTS-99L2 (both from Texaco, special noncommercial designations).

Samples without added zinc were Ferrodiesel 597 (Repsol-YPF), Gascon Supreme Plus MVI 40 (Lyondel), Viscodis 220 (ANCAP) and CAD-43 (Petrobras).

### 2.4. Methods

Standard solutions were prepared by exact dilution by weight of the Conostan standard with BO-75 base oil.

Samples were introduced to the flow system without further processing.

For purposes of validation, analytical results obtained with the proposed method were compared with those obtained by an ASTM batch flame AAS method [4]. In this method samples were diluted by weight with metal-free kerosene and measured by AAS with a nitrous oxide-acetylene flame.

### 3. VALIDATION

Linearity, accuracy, precision, detection limit (LOD), quantification limit (LOQ), carryover, stability, and sampling frequency were the figures of merit considered.

### 3.1. Linearity

Linearity was assessed from calibration curves by means of the least-squares method (5 concentration levels).

### 3.2. Accuracy and precision

Accuracy was evaluated by comparing the mean values of the results obtained by the proposed method with those obtained by the standard ASTM method. Student's $t$ test for the mean values was used at the 95% confidence level.

Precision was assessed from the results of these determinations.

### 3.3. Carryover

The possible existence of memory effects between successive injections was studied, in the low-concentration system by injecting a Viscodis 220 oil sample (containing about 8.7 ppm Zn) followed by injection of BO-75 base oil blank.

For the high-concentration system, Superdiesel 40 oil (0.14% (w/w) Zn) and BO-75 were used for this purpose.

### 3.4. Stability

In order to assess the stability of the system during long runs, a sample of Superdiesel 40 oil containing about 0.14% (w/w) Zn was injected 30 times during a period of about 60 minutes.

### 4. RESULTS AND DISCUSSION

### 4.1. Injection technique

Contemporary flow-injection analysis relies on the use of six-port (or similar) injection valves for the introduction of the sample into the carrier stream. However in the early times of FIA, syringes were used for this purpose as attested by the first paper describing the technique [16].

When using injection valves, a sampling loop defines the volume of sample to be injected. In automated systems, a peristaltic pump is usually employed to fill this loop.

The difficulties associated with the handling of highly viscous samples such as lubricating oils are obvious. Peristaltic pumps, that are one of the usual pumping devices in automation, are of little value for handling this kind of samples, even for a simple task such as loading a sampling loop. In the first place, the flow rate is not independent from the viscosity (which in turn depends on the temperature and oil type). Besides, the pumping tubes have a high dead volume. When changing samples, it is extremely difficult to clean the pumping tubes and the sampling loop from the previous sample, if memory effects are to be avoided. This results in lengthy operations and low sample throughput.

In order to circumvent this problem, preliminary experiments were carried out trying to use the peristaltic pumps to generate negative pressure (i.e., sipping the sample through the sampling loop), but this approach failed when applied to lubricating oils.

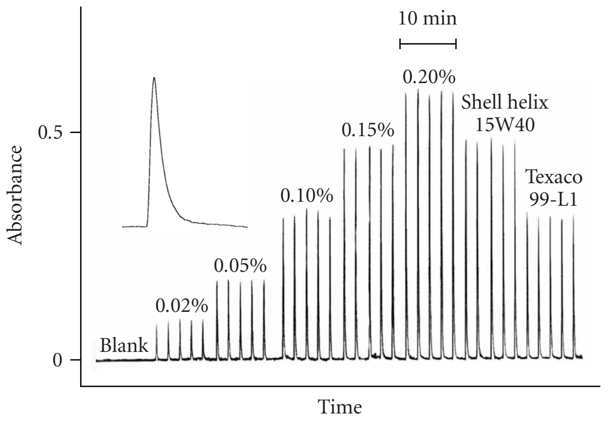

Figure 3: Recording of the signal corresponding to a calibration curve and two samples (concentrations in %(w/w)). Upper left angle: expanded trace of a typical peak.

In previous work [14], a motorised syringe has been successfully applied to the automated analysis of lubricating oils for wear metals. The piston motion demonstrated to be quite effective in displacing the oil out of the barrel, even with dirty samples, and good analytical precision could be achieved. Motorisation avoids the uncertainties associated with manual injection and is amenable to automation. Therefore, this approach was applied in the present work.

The use of a motorised syringe as injection device for oil samples deserves some comment. When the injection is performed via an injection valve fitted with a sampling loop, the sample is injected as a homogeneous "plug" which is subsequently dispersed in the carrier stream. When low viscosity samples are injected rapidly with a syringe as in early FIA work [16], the injected sample is supposed to displace an equal volume of carrier, resulting then in the injection of a sample "plug" which, once dispersed, will produce a mixing pattern and a peak shape similar to those obtained with loop injection. On the other hand, the slow injection of a viscous sample (such as an oil sample) produces a laminar flow pattern where both sample and carrier flow alongside with little mutual interaction unless some kind of mixing device is used to provide a vigorous mixing action.

In the present system, the injection had to be inherently slow because the viscosity of the sample would otherwise produce unacceptably high pressures inside the syringe barrel and other parts of the system. In this instance, the mixing action was provided by the packed mixing reactor ("MR" in Figure 1). Figure 3 shows the recording corresponding to a calibration curve and samples. The peak shapes obtained do not differ significantly from typical FIA peaks, suggesting that the radial mixing action of the mixing reactor is highly efficient. This was attributed to the random nature of the packing, which forces the flow into complex patterns with multiple direction changes.

### 4.2. Linearity

A second-degree model provided the best fit. Regression equation was

$$h = -4 \cdot 10^{-8}C^2 + 0.0004C \quad (r^2 = 0.996), \qquad (1)$$

where $h$ is the peak height (absorbance) and $C$ is the concentration (ppm). Linear regression showed slightly worse fit ($r^2 = 0.993$), thus the second-degree model was chosen for calibration.

### 4.3. Accuracy and precision

Analysis performed on the commercial samples showed no significant differences between the two methods at the 95% confidence level. Results can be seen in Table 1. Precision attained can also be seen in this table.

Precision (RSD%) obtained with the HCS was always better than 5% and usually better than 2%. This was considered adequate for the analysis of this kind of samples. Precision is limited partly by the uncertainty in the injected volume. This in turn is affected by the resolution of the syringe. Considering the mechanical resolution of the stepping motor used, the lab-made syringe employed had a volumetric resolution of 2 $\mu$L, which is 1% of the sample volume. It is predictable that better results could be obtained by using a motorised syringe with higher resolution.

Determinations carried out with the LCS exhibited higher dispersion, but this could be predicted given the low concentrations involved and hence the low absorbance values found.

### 4.4. Detection and quantification limits

For the LCS, LOD ($3\sigma$) was 0.08 ppm, and LOQ ($10\sigma$) was 0.28 ppm. For the HCS, LOD ($3\sigma$) was 33 ppm, and LOQ ($10\sigma$) was 110 ppm.

*Carryover*

In preliminary experiments, when injected repeatedly the BO-75 base oil blank, no signal was detected in either LCS or HCS systems. For the LCS, when injecting the BO-75 blank after the Viscodis 220 oil sample, no signal was detected

When the same experiment was carried out in the HCS system with a Superdiesel 40 oil sample, the signal corresponding to the blank was less than 2.5% when compared with the signal of the Superdiesel 40 oil (Figure 4). This is the maximum extent of memory effect to be expected under the conditions established.

*Stability*

Figure 5 shows the plot of the 60-minute run for the HCS. It can be observed that neither baseline nor response (peak height) varied significantly in that period, suggesting that the system is robust in long-term runs.

*Sampling frequency*

Under the conditions established, the experimental sampling frequency was 30 samples per hour for the HCS, and around 40 samples per hour for the LCS.

Table 1: Comparison of the results obtained in the determination of zinc in commercial lubricating oil samples by means of the automated FIA system (proposed method) and the ASTM method (reference method). Mean values expressed either in ppm or %(w/w). n denotes number of repetitions, $t_{exp}$ experimental value of Student's *t* statistic, $t_{table}$ calculated value of Student's *t* statistic taken from the *t* distribution at the 95% confidence level, and (*) special noncommercial designations.

| Samples without Zn addition | Proposed method | | | Reference method | | | | | |
|---|---|---|---|---|---|---|---|---|---|
| Brand name | Mean (ppm) | RSD% | n | Mean (ppm) | RSD% | n | $t_{exp}$ | $t_{table}$ | Result |
| Repsol-YPF Ferrodiesel 597-1 | 0.9 | 12.5 | 5 | 0.9 | 5.0 | 5 | 0.33 | 2.31 | ✓ |
| Repsol-YPF Ferrodiesel 597-2 | 0.6 | 14.3 | 4 | 0.6 | 7.5 | 5 | 0.79 | 2.37 | ✓ |
| ANCAP Viscodis 220 | 8.7 | 4.1 | 5 | 8.3 | 1.4 | 5 | 1.96 | 2.31 | ✓ |
| Petrobras CAD-43 | 2.5 | 3.8 | 4 | 2.5 | 3.6 | 5 | 0.09 | 2.37 | ✓ |
| Lyondel Gascon MVI 40 | 1.1 | 20.2 | 5 | 1.1 | 1.6 | 5 | 0.10 | 2.31 | ✓ |
| | | | | | | | | | |
| Samples with Zn addition | Proposed method | | | Reference method | | | | | |
| Brand name | Mean (%) | RSD% | n | Mean (%) | RSD% | n | $t_{exp}$ | $t_{table}$ | Result |
| Shell Helix 15W40 | 0.154 | 2.0 | 5 | 0.164 | 4.6 | 5 | 1.05 | 2.31 | ✓ |
| Shell Tellus 46 | 0.036 | 4.7 | 5 | 0.035 | 2.5 | 5 | 0.15 | 2.31 | ✓ |
| Shell Rimula D 30 | 0.080 | 1.0 | 5 | 0.078 | 3.9 | 5 | 0.52 | 2.31 | ✓ |
| ANCAP Superdiesel 40 | 0.141 | 1.4 | 5 | 0.148 | 3.7 | 5 | 0.94 | 2.31 | ✓ |
| Texaco GTS-99L1 (*) | 0.097 | 2.7 | 5 | 0.113 | 6.0 | 5 | 1.85 | 2.31 | ✓ |
| Texaco GTS-99L2 (*) | 0.110 | 1.7 | 5 | 0.121 | 4.5 | 5 | 1.58 | 2.31 | ✓ |

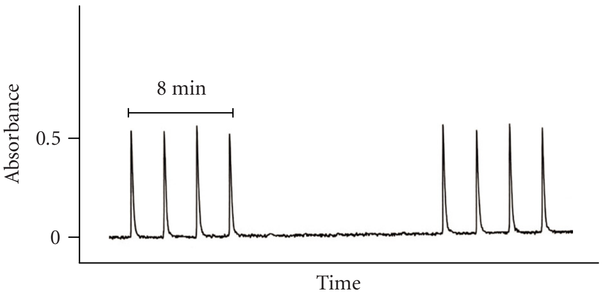

Figure 4: Signal recording corresponding to the test for memory effect. Four injections of Superdiesel 40 oil (Zn contents about 0.14% w/w), four injections of blank oil (BO-75), and four injections of Superdiesel 40.

### 5. CONCLUSIONS

The method of injecting the sample by means of a motorised syringe, associated with a packed mixing reactor has demonstrated to be a useful tool for the automation of the analysis of lubricating oil samples in a flow-injection system. These highly viscous samples can be successfully injected in a stream of organic solvent and dispersed into this solvent in a reproducible way.

When this system was applied to the automation of the determination of organometallic additives by AAS, satisfactory results were obtained, which compare favourably with those obtained with a reference method.

It is concluded that the use of this kind of injection in flow-injection systems is a promising tool that extends the capabilities of flow-injection analysis to highly viscous samples.

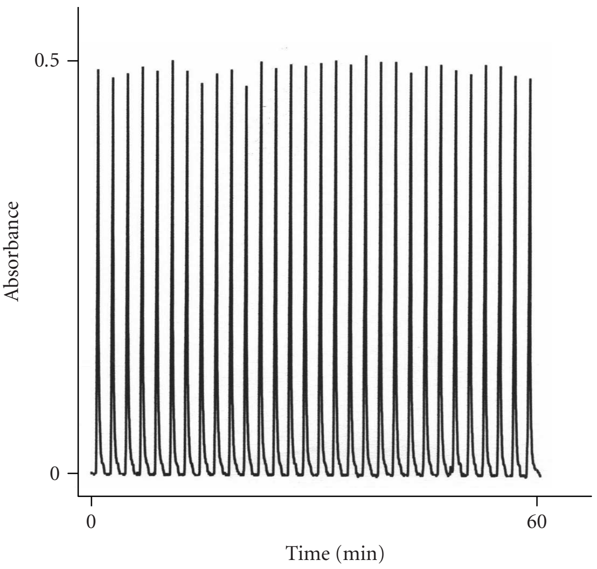

Figure 5: Signal recording corresponding to the stability test. A commercial sample (Superdiesel 40 oil) containing about 0.14% Zn was injected 30 times in a 1-hour period.

### ACKNOWLEDGMENTS

The authors thank Alexandra Sixto and Leticia Vázquez for the enthusiastic cooperation with the AAS measurements. Partial financial support from UDELAR/CSIC and UNDP/PEDECIBA (Program URU/97/016) is gratefully acknowledged as well.

### REFERENCES

[1] T. V. Liston, "Engine lubricant additives, what they are and how they work," *Lubrication Engineering*, vol. 48, no. 5, pp. 389–397, 1992.

[2] ASTM, "Standard Test Method for Determination of Additive Elements, Wear Metals, and Contaminants in Used Lubricating Oils and Determination of Selected Elements in Base Oils by Inductively Coupled Plasma Atomic Emission Spectrometry," in *1992 Book of ASTM Standards, Section 5, V. 5.03*, American Society for Testing and Materials, Philadelphia, Pa, USA, 1992, Method D - 5185-91.

[3] ASTM, "Standard Test Method for Determination of Additive Elements in Lubricating Oils by Inductively Coupled Plasma Atomic Emission Spectrometry," in *1992 Book of ASTM Standards, Section 5, V. 5.03*, American Society for Testing and Materials, Philadelphia, Pa, USA, 1992, Method D - 4951-89.

[4] ASTM, "Standard Test Method for Determination of Additive Elements in Lubricating Oils by Inductively Coupled Plasma Atomic Emission Spectrometry," in *1992 Book of ASTM Standards, Section 5, V. 5.03*, American Society for Testing and Materials, Philadelphia, Pa, USA, 1992, Method D - 4628-86.

[5] M. de la Guardia and A. Salvador, "Flame atomic absorption determination of metals in lubricating oils: a critical review," *Atomic Spectroscopy*, vol. 5, pp. 150–155, 1984.

[6] Z. Wittmann, "Direct determination of calcium, magnesium and zinc in lubricating oils and additives by atomic-absorption spectrometry using a mixed solvent system," *Analyst*, vol. 104, pp. 156–160, 1979.

[7] P. K. Hon, O. W. Lau, and C. S. Mok, "Direct determination of metals in lubricating oils using atomic-absorption spectrometry and aqueous inorganic standards," *Analyst*, vol. 105, p. 919, 1980.

[8] V. Cerdà and G. Ramis, *An Introduction to Laboratory Automation*, Wiley, New York, USA, 1990.

[9] P. B. Stockwell, *Automatic Chemical Analysis*, Taylor and Francis, London, UK, 2nd edition, 1996.

[10] J. Ruzicka and E. Hansen, *Flow Injection Analysis*, Wiley, New York, USA, 2nd edition, 1989.

[11] M.P. Granchi, J. A. Biggerstaff, L. J. Milliard, and P. Grey, "Use of a robot and flow injection for automated sample preparation and analysis of used oils by ICP emission spectrometry," *Spectrochimica Acta B*, vol. 42, pp. 169–180, 1987.

[12] R. M. Borja, A. Salvador, M. de la Guardia, J. L. Burguera, and M. Burguera, *Química Analítica*, vol. 8, p. 241, 1989.

[13] J. L. Burguera, R. A. de Salager, M. Burguera, et al., "On-line emulsification of lubricating oils in a flow-injection system for chromium determination by electrothermal atomic absorption spectrometry," *Journal of Analytical Atomic Spectrometry*, vol. 15, no. 5, pp. 549–555, 2000.

[14] G. Pignalosa and M. Knochen, "Determination of wear metals in lubricating oils using flow injection AAS," *Atomic Spectroscopy*, vol. 22, pp. 250–257, 2001.

[15] D. A. Whitman and G. D. Christian, "Cascade system for rapid on-line dilutions in flow-injection analysis," *Talanta*, vol. 36, no. 1-2, pp. 205–211, 1989.

[16] J. Ruzicka and E. H. Hansen, "Flow injection analyses : Part i. A new concept of fast continuous flow analysis," *Analytica Chimica Acta*, vol. 78, no. 1, pp. 145–157, 1975.

